# Trends in the Prevalence of Orofacial Clefts Among Hispanic Infants in the Rio Grande Valley of Texas (1997–2018)

**DOI:** 10.7759/cureus.84899

**Published:** 2025-05-27

**Authors:** Yossef Alsabawi, Aaron Dadzie, Kelsey Baker

**Affiliations:** 1 Medical Education, The University of Texas Rio Grande Valley School of Medicine, Edinburg, USA; 2 Neuroscience, The University of Texas Rio Grande Valley, Edinburg, USA

**Keywords:** cleft lip & palate, health disparities in rio grande valley, oro-facial congenital anomalies, prevalence rate, rio grande valley

## Abstract

Background

Orofacial clefts (OFCs), including the cleft lip with or without cleft palate (CLP) and cleft palate alone (CP), represent significant congenital anomalies with implications for feeding, speech, and psychosocial development. The Rio Grande Valley (RGV) of Texas, a predominantly Hispanic region that faces socioeconomic and healthcare challenges, may have a distinct OFC profile compared to non-border counties (NBCs) of Texas.

Methods

Prevalence data with 95% confidence intervals for OFCs from 1997 to 2018 were obtained from the Texas Department of State Health Services (DSHS). Definitions provided by the DSHS were adhered to, including only definite diagnoses. Given the large sample sizes and annual prevalence, a two-tailed z-score analysis was utilized to compare the RGV with NBCs, assuming normal approximation per the central limit theorem. Hispanic mothers were selected as the study population to minimize confounding by ethnicity, as the RGV has a predominantly Hispanic population.

Results

Overall, the prevalence of CLP among children born to Hispanic mothers in the RGV was higher than in NBCs (*p* = 0.26). The prevalence of CLP from 2008 to 2018 was significantly higher in the RGV than in NBCs (*p *= 0.006). From the first decade (1997-2007) to the second (2008-2018), the prevalence of CLP decreased significantly in NBCs (*p* = 0.016) and increased in the RGV (*p* = 0.06). No significant differences in the prevalence of CP were found across regions or time.

Conclusion

These findings suggest divergent temporal trends between CLP in the RGV versus NBCs. Therefore, there is a need for targeted public health interventions, given potential reporting limitations. Enhanced surveillance, improved diagnostic reporting (particularly in medically underserved and undocumented populations), and further research into environmental and socioeconomic determinants are warranted.

## Introduction

Orofacial clefts (OFCs) are among the most common congenital anomalies, affecting approximately 10 per 10,000 live births in the United States [1]. These defects can lead to significant feeding difficulties, speech impairments, and psychosocial challenges that require coordinated surgical and rehabilitative care [2,3].

The Rio Grande Valley (RGV) of Texas, a region characterized by high poverty rates, limited healthcare resources, and a predominantly Hispanic population, presents unique public health challenges. According to the U.S. Census Bureau data, the RGV exhibits some of the highest poverty and uninsured rates in the state, factors that have been linked to reduced access to prenatal care and delayed diagnoses [4]. Additionally, the RGV is home to a large population of undocumented migrants, although exact numbers cannot be determined. Although previous studies have addressed ethnic disparities in OFC nationally, few have explicitly focused on the RGV [5]. The present study leverages Texas Department of State and Health Services (DSHS) data to analyze temporal trends in OFC prevalence among infants born to Hispanic mothers.

## Materials and methods

Study design and data source

We conducted an ecological, population-based surveillance study using aggregated, de-identified data from the Texas Department of State Health Services (DSHS) Birth Defects Epidemiology and Surveillance Branch for the years 1997 through 2018. These data were provided upon request through personal communication. The Texas Birth Defects Registry (TBDR) collects data by reviewing hospital logs, discharge lists, and other medical records of infants born with birth defects. These data are utilized to track and characterize statewide trends in birth defect rates. The dataset included annual counts and calculated prevalence rates (per 10,000 live births) with corresponding 95% confidence intervals (CIs) for OFCs, stratified by county of maternal residence. Cells with fewer than five cases in geographic units smaller than a county were suppressed by DSHS to protect privacy.

Study setting and population

Our primary region of interest was the RGV, comprising Cameron, Hidalgo, Starr, and Willacy counties. For comparison, we used aggregated data from all other Texas counties that do not border Mexico. To minimize confounding by ethnicity, analyses were restricted to infants born to mothers who self-identified as Hispanic. Only definite diagnoses of cleft lip with or without cleft palate (CLP) and isolated cleft palate (CP) were included, as defined by the DSHS clinical criteria. We excluded any records lacking county of residence or maternal ethnicity information, as well as suppressed data points (<10 cases) that could not be disaggregated.

Case definitions

We included live-born singleton infants born between January 1, 1997, and December 31, 2018, whose mothers resided in one of the four counties of the RGV of Texas or any non-border counties of Texas. Only cases in which maternal ethnicity was recorded as Hispanic were considered. Infants were included if they had a definite diagnosis of cleft lip with or without cleft palate (CL ± P; DSHS codes 749.10-749.29) or isolated cleft palate (CP; DSHS codes 749.00-749.09). Infants with identifiable syndromic or chromosomal conditions, based on the DSHS linkage, were excluded to focus the analysis on non-syndromic presentations.

Ethical approval

Data were provided in aggregate form with no individual identifiers, and small-cell suppression ensured privacy. This project was determined to be exempt from IRB review by the Institutional Review Board of the University of Texas Rio Grande Valley (IRB-23-0323).

Statistical analysis

Prevalence rates were compared between the RGV and non-border counties using two‐tailed z‐tests for differences between proportions, based on the supplied 95% CIs and assuming approximate normality by the central limit theorem. We conducted subgroup analyses by decade (1997-2007 vs. 2008-2018) to assess temporal trends. A p-value < 0.05 was considered statistically significant. All analyses were performed in JupyterLab (version 4.2.5).

## Results

Overall prevalence (1997-2018)

**Figure 1:**
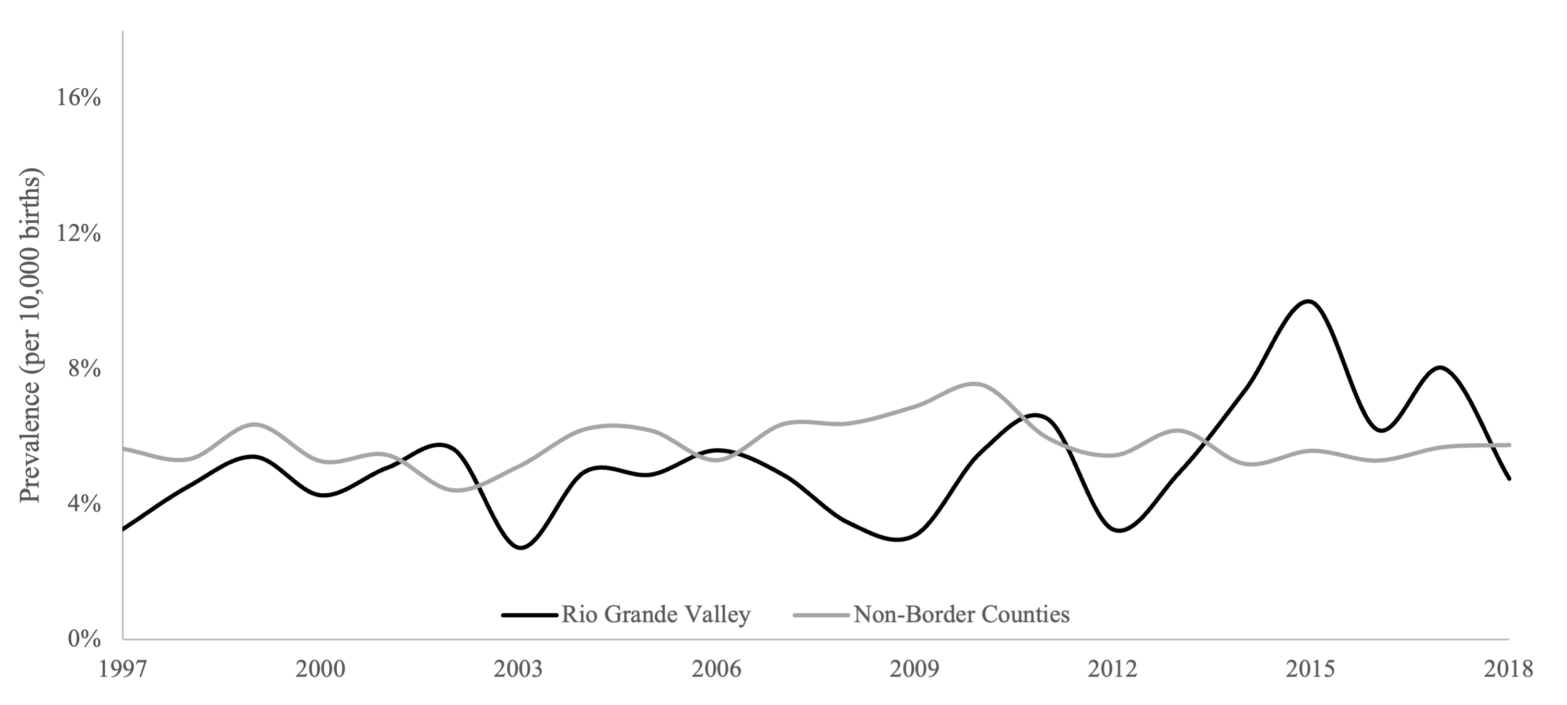
Prevalence of cleft palate alone (1997-2018).

**Figure 2:**
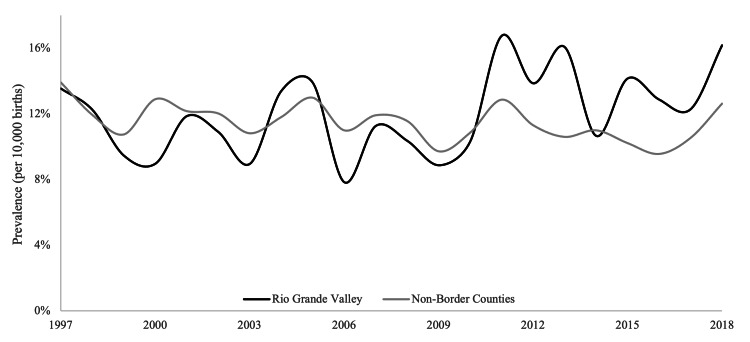
Prevalence of cleft lip with or without cleft palate (1997-2018).

The overall prevalence of CP in the RGV was lower than in non-border counties. This difference approached but did not reach statistical significance (*p* = 0.07). CLP prevalence was higher in the RGV but did not differ significantly between regions (*p* = 0.26). Results are summarized in Table 1 and Figures 1, 2 (Unpublished data: Texas Department of State Health Services, March 3, 2023).

**Table 1:** Prevalence of orofacial clefts (1997-2018). OFC = orofacial cleft; CP = cleft palate alone; CLP = cleft lip with or without palate; RGV = Rio Grande Valley of Texas; NBC = non-border counties of Texas.

	RGV		NBC			
OFC type	Cases	Prevalence (95% CI)	Cases	Prevalence (95% CI)	Z-score	*p-*value
CP	276	5.18 (4.57 - 5.79)	1739	5.82 (5.55 - 6.10)	-1.82	0.07
CLP	638	11.97 (11.04 - 12.90)	3406	11.41 (11.02 - 11.79)	-1.12	0.26

Intra-regional comparisons

In the RGV, CP prevalence increased slightly from the first decade to the second (*p* = 0.11), a trend mirrored by non-border counties (*p* = 0.18). Similarly, an increased CLP prevalence was noted in the RGV from the first to the second decade (*p* = 0.06), whereas in non-border counties, CLP prevalence significantly decreased (*p* = 0.016). Results are summarized in Tables 2, 3 (Unpublished data: Texas Department of State Health Services, March 3, 2023).

**Table 2:** Time-trend analysis of the Rio Grande Valley. OFC = orofacial cleft; CP = cleft palate alone; CLP = cleft lip with or without palate.

	1997-2007		2008-2018			
OFC type	Cases	Prevalence (95% CI)	Cases	Prevalence (95% CI)	Z-score	*p-*value
CP	126	4.68 (3.90 - 5.57)	150	5.69 (4.82 - 6.68)	-1.62	0.11
CLP	299	11.10 (9.88 - 12.43)	339	12.86 (11.53 - 14.30)	-1.86	0.06

**Table 3:** Time-trend analysis of non-border counties. OFC = orofacial cleft; CP = cleft palate alone; CLP = cleft lip with or without palate.

	1997-2007		2008-2018			
OFC type	Cases	Prevalence (95% CI)	Cases	Prevalence (95% CI)	Z-score	*p-*value
CP	777	5.62 (5.23 - 6.03)	962	6.00 (5.63 - 6.39)	-1.35	0.18
CLP	1647	11.92 (11.35 - 12.51)	1759	10.97 (10.46 -11.49)	-2.42	0.016

Inter-regional comparisons

Between 1997 and 2007, CP prevalence was lower in the RGV than in non-border counties (*p* = 0.05). During the same decade, CLP prevalence was similarly lower in the RGV (*p* = 0.26). From 2008 to 2018, the RGV continued to have a lower CP prevalence without a statistically significant difference (*p* = 0.55). However, the RGV exhibited a significantly higher CLP prevalence during this period than in non-border counties (*p* = 0.006). Results are summarized in Table 4 (Unpublished data: Texas Department of State Health Services, March 3, 2023).

**Table 4:** Regional analysis of orofacial clefts by decade. OFC = orofacial cleft; CP = cleft palate alone; CLP = cleft lip with or without palate; RGV = Rio Grande Valley of Texas; NBC = non-border counties of Texas.

	RGV		NBC			
OFC type	Cases	Prevalence (95% CI)	Cases	Prevalence (95% CI)	Z-score	*p*-value
CP						
1997-2007	126	4.68 (3.90 - 5.57)	777	5.62 (5.23 - 6.03)	-1.92	0.05
2008-2018	150	5.69 (4.82 - 6.68)	962	6.00 (5.63 - 6.39)	-0.6	0.55
CLP						
1997-2007	299	11.10 (9.88 - 12.43)	1647	11.92 (11.35 - 12.51)	-1.13	0.26
2008-2018	339	12.86 (11.53 - 14.30)	1759	10.97 (10.46 -11.49)	-2.51	0.006

## Discussion

Summary of key findings

This analysis of Texas birth defects surveillance data from 1997 to 2018 investigated OFC prevalence trends among Hispanic infants in the RGV compared to non-border counties. The study identified distinct patterns for cleft lip with or without cleft palate (CLP) versus cleft palate alone (CP). Notably, while overall CLP prevalence did not differ significantly between the groups across the entire period, a significant divergence occurred between 2008 and 2018, with CLP prevalence becoming significantly higher among RGV Hispanic infants compared to their non-border counterparts (p = 0.007). Within the RGV, there was also a borderline statistically significant increase in CLP prevalence when comparing 1997-2007 to 2008-2018 (p = 0.063), contrasting with a non-significant decrease in non-border counties. Conversely, CP prevalence trended non-significantly lower in the RGV compared to non-border counties overall (p = 0.059), with non-significant increases observed in both regions over time (Unpublished data: Texas Department of State Health Services, March 3, 2023). The primary implication is the identification of a significantly elevated and potentially increasing CLP prevalence, specifically among RGV Hispanic infants in the more recent decade, highlighting a localized public health concern.

Contextualization and potential factors

These findings, particularly the diverging CLP trend, warrant comparison with broader OFC epidemiology. Texas-wide data (1999-2014) showed overall non-syndromic CLP prevalence at 8.3 and CP at 3.0 per 10,000 live births, with Hispanic ethnicity associated with similar CLP but lower CP rates compared to non-Hispanic White infants, aligning directionally with the non-significant lower CP trend observed in the RGV [6]. March of Dimes data (2014-2017) reported a Texas Hispanic CLP rate of 7.8 per 10,000 [7]. National studies present varied findings regarding Hispanic OFC prevalence compared to other groups, sometimes reporting higher overall rates but often lower CP rates compared to the non-Hispanic White population [8,9]. The significantly higher CLP rate found in this study for RGV Hispanic infants (2008-2018) suggests a localized elevation potentially exceeding general US Hispanic trends. This is particularly concerning given findings that Hispanic individuals in the US may face higher mortality associated with CLP [10].

The RGV's unique context, predominantly Hispanic population, high poverty rates, medically underserved status, and environmental concerns (pesticides, industrial pollution, water quality), likely contributes [11]. While direct links between specific RGV environmental exposures and OFCs require more research, the region has known exposures (e.g., pesticide metabolites and polycyclic aromatic hydrocarbons ) and historical birth defect concerns, particularly regarding neural tube defects [12,13]. The multifactorial etiology of OFCs involves interactions between genetics and maternal/environmental factors [2,14]. Established risk factors relevant to the RGV include maternal diabetes and obesity (regionally prevalent), smoking, and potentially nutritional factors [14,15]. Lower socioeconomic status, also prevalent in the RGV, is associated with OFC risk, potentially mediated via nutrition, stress, exposures, and healthcare access [16]. Significant challenges in accessing prenatal care in the RGV can hinder the management of maternal risks and nutritional counseling [13].

Limitations

This study has several important limitations. The use of aggregated surveillance data prevented adjustment for individual-level confounders such as maternal age, socioeconomic status, smoking, diabetes, and body mass index. These risk factors may vary regionally and influence the risk of OFCs. Case ascertainment may be incomplete, as the Texas registry includes live births and fetal deaths in state facilities but may miss out-of-state terminations, home births, and early stillbirths. Suppression of small cell counts in county-level data can introduce bias in less populous areas and obscure smaller spatial clusters, while also limiting urban-rural comparisons within counties. This study’s focus on only non-syndromic OFCs reduces generalizability and may obscure true differences in OFC trends, particularly if the distribution of syndromic cases differs by location. Cross-border healthcare use is common in the RGV, and infants born in Mexico or to temporarily non-resident mothers may not be recorded in Texas records, leading to underreporting in the region of interest. Finally, regional comparisons are subject to ecological fallacy, as group-level differences may not accurately reflect individual risk.

Public health implications and future directions

The findings signal a need for focused public health attention in the RGV, particularly given regional healthcare access challenges that can impede the complex, multidisciplinary care needed for OFCs. Robust primary prevention efforts addressing modifiable risks (maternal health, nutrition, smoking cessation) and enhancing access to quality prenatal care are crucial. Strengthening referral systems connecting families to support services like early childhood intervention (ECI) is also vital.

Future research should prioritize identifying the specific drivers of the RGV's increasing CLP trend. This requires etiologic studies using individual-level data on environmental exposures, detailed nutrition, maternal health, genetics, and socioeconomic factors. Research must differentiate between cleft subtypes (syndromic/non-syndromic, isolated/non-isolated). Enhanced surveillance, potentially via binational collaboration and improved data linkage, is needed to better capture the true prevalence in this border region. Evaluating culturally tailored interventions and investigating barriers to accessing specialized cleft care, possibly leveraging insights from ongoing research like the Birth Defects Study To Evaluate Pregnancy Exposures (BD-STEPS), are also key priorities.

## Conclusions

This study reveals that between 2008 and 2018, Hispanic infants born in the RGV experienced a significantly higher prevalence of CLP compared to those in non-border counties, while CP prevalence remained lower. Although limited by aggregated data and potential underreporting, these findings highlight regional disparities and the need for improved healthcare access and reporting systems. Tailored public health initiatives and further research into underlying risk factors are essential to mitigate the burden of OFCs in the RGV.
